# Daily School Physical Activity Improves Academic Performance

**DOI:** 10.3390/sports8060083

**Published:** 2020-06-04

**Authors:** Jesper Fritz, Marcus E. Cöster, Björn E. Rosengren, Caroline Karlsson, Magnus K. Karlsson

**Affiliations:** Clinical and Molecular Osteoporosis Research Unit, Department of Orthopedics and Clinical Sciences, Lund University, 20502 Malmö, Sweden; marcus.coster@med.lu.se (M.E.C.); bjorn.rosengren@med.lu.se (B.E.R.); Caroline.i.karlsson@telia.com (C.K.); Magnus.karlsson@med.lu.se (M.K.K.)

**Keywords:** physical activity, children, academic performance, physical education

## Abstract

Physical activity (PA) may improve brain development, cognition, concentration and academic performance. In this prospective controlled intervention study, we increased the level of PA in 338 children aged 6–8 years at study start, from the Swedish standard of 60 min per week to 200 min per week (40 min daily). The intervention continued in all nine compulsory school years until the students graduated between 2007–2012. All other 689,881 Swedish children who graduated the same years were included as a control group. We registered at graduation eligibility rate for upper secondary school and the final grade score (from 0 to 320 grade points). We also registered the same end points in the 295 students in the index school and in all other 471,926 Swedish students who graduated in 2003–2006, that is, those who graduated before the intervention study started. Before the intervention, academic performance was similar among children in the index school as for all other Swedish boys and girls. With the intervention, the eligibility rate increased for boys in the index school by 7.3 percentage points and the mean grade scores by 13.3 points. This should be compared with a decrease of 0.8 percentage points in eligibility rate and an increase by 2.7 points in grade score in other Swedish boys. No changes were seen for intervention girls, neither in eligibility rates or grade scores. By introducing daily school-based PA in compulsory school, more boys would probably reach the eligibility rate for higher education.

## 1. Introduction

Lifestyle habits in childhood tend to follow the individual into adulthood [1,2,3]. Therefore, it is important in early years to already influence children to adopt healthy lifestyle habits, such as a physically active lifestyle, good sleep and good dietary habits [2]. Such habits are supported in school in Sweden, for example throughout repeated general information sessions by school nurses and teachers, not only to the students but also to parents or guardians. Large observational studies have also found that individuals with higher education, such as a university degree, have healthier lifestyles, higher self-esteem, are less likely to suffer from chronic diseases and have lower mortality rates than individuals without such education [3,4,5]. Society ought, therefore, to raise the educational level in society.

The proportion of Swedish children who graduate from the 9th and final year of compulsory schooling without eligibility for upper secondary school programs has increased during recent decades [6,7]. The proportion of eligible students was only 86% in 2015, the lowest proportion since 1998 [6]. The decrease is a paradox since researchers claim that 100% of Swedish students have the potential and capacity to reach the goals required for a passing grade in all school subjects [8]. School results, with reading performance, mathematics and performance in science as end point variables, have in the Program for International Student Assessment (PISA) been reported to decrease in Western countries [9]. Declining academic school performance should, therefore, be considered an international issue and the question is how to change this. One approach may possibly be to increase the level of physical activity (PA), as high level of PA is associated with superior intellectual performance [10,11]. For example, one study showed that 2 extra weekly school PA classes of 30–45 min including ‘‘play and motion’’ activities was in girls associated with higher likelihood to pass national tests in Swedish (odds ratio 5.7) and Mathematics (odds ratio 3.2) [11]. Another study showed that among 1271 students from urban Santiago, Chile, 4 hours per week of scheduled exercise was associated with having System for the Assessment of Educational Quality (SIMCE) composite z-scores ≥50th percentile (odds ratio 2.3) and ≥75th percentile (odds ratio 2.1) [10]. Despite this knowledge, there has been a tendency to reduce physical education (PE) during recent decades in favor of academic subjects [12]. Some reports have, therefore, postulated that the decrease seen in school results could at least partly be referred to the lower exposure to PA [13,14]. 

Hypothetically, increasing PE in school may, therefore, reverse the negative trend of school performance [13,15]. Recently this has been shown in a smaller pilot study at a regional level in southern Sweden [15]. The study found that daily physical activity during the nine compulsory school years was associated with a higher grade score. To our knowledge, no other prospective population-based intervention study has addressed the question by including all children within a country as control cohort. It has also been postulated that boys and girls could respond differently to such an intervention, since boys and girls respond differently to PA during growth [10] and since boys have a greater potential to improve, with their school results lower compared to girls [16]. Few studies have addressed this hypothesis and have either included small samples, been short-term, used specific groups or used different surrogate end points for academic performance [17,18,19,20,21], possibly explaining the divergent conclusions and lack of consensus on the effect of increased PA on academic school performance. A systematic review from 2018 describes a strong need for high-quality evidence with adequate control groups and end points [22]. Previous studies have shown increased PE to have positive effects on bone mass, muscle strength and fracture risk [4,23], but a positive effect on academic performance and thereby increased proportion of students eligible for higher education may have an even larger impact on overall health.

Therefore, we conducted a sex specific 9 year, population-based, prospective controlled, PA intervention study during growth. Our aim was to evaluate if daily school PA induced higher eligibility rate and higher final grade score than having school PA 1–2 sessions per week. Our hypothesis was that daily physical activity would be associated with increased eligibility rate and higher final grade score than having 1–2 sessions per, but with more obvious effect in boys than in girls, as boys have lower eligibility and lower grade scores than girls, and by this greater potential for improvement.

## 2. Materials and Methods

### 2.1. The Malmo Pediatric Osteoporosis Prevention (POP) Study

The Malmo Pediatric Osteoporosis Prevention (POP) study is a prospective controlled PA intervention study that evaluates the effects of increased school-based physical education (PE) on health and school performance. The study design has previously been presented in detail [15,24]. In summary, one school (index school) was invited to increase PE from 1–2 lessons per week to one daily lesson per school week. We chose to conduct the intervention in school since this is the only arena that reaches all children and PE is a compulsory school subject in all Swedish schools, and all children thus had to participate. The children were 6–8 years old at study start [24] and in this report we followed the children for nine years (the entire compulsory Swedish elementary school) until they were 15–17 years old. At that time, we evaluated school performance by eligibility rate to upper secondary school and overall grade score. The study was approved by the ethics committee of Lund University (LU 453-98; 1998-09-15), conducted according to the Declaration of Helsinki and was registered as a clinical trial (ClinicalTrials.gov.NCT00633828). Both oral and written signed consents were collected from parents or guardians and students before study start.

From school start in the index school, we increased the amount of PE per week from the Swedish standard of 60 min to 200 min given as one lesson of 40 min per school day. These lessons were scheduled as all other lessons and were given within the regular school curriculum. The PA included moderate to intense activities from the regular Swedish PE curriculum, such as gymnastics, team sports, dancing, running, jumping, and playing activities. We did not register types of activities included in the school PE. We did not measure the PA level that each student used at the PE lessons, only the duration of the activity. However, all students participated at least with the minimal required level, since all students received a passing grade in the subject PE. The intervention was ongoing in all children who started school during the years 1998–2003 and throughout all nine compulsory school years until they finished 9th grade in the years 2007–2012. The PE classes were led by the regular teachers and followed the regular PE curriculum. This curriculum included activities such as ball games, running, jumping, and playing, on a moderate to vigorous intensity. There were no extra PE classes during weekends or during the 15 weeks of annual school holidays. The school followed the regular national school curriculum in all other subjects.

### 2.2. Probands

All 234 boys and 190 girls who started grade one in the index school years 1998–2003 were included in the intervention from grade 1. The children (49 boys and 37 girls) who left the index school during the study period (and thus did not complete all nine years of intervention)*,* in almost all cases due to relocation of the family to another region in the city, another part of the country or abroad, were excluded. Since there at baseline were no differences in age, gender, weight, or body mass index when comparing those who left with those who stayed, the risk of selection bias seems minor (data not shown). Thus, 185 boys and 153 girls remained in the intervention group in this report.

To be able to identify any changes within the index school from the period before the intervention was initiated and after the intervention was initiated (the study period), we registered the same end points in children who started grade one in the index school during years 1994–1997 (175 boys and 181 girls). We excluded 25 boys and 41 girls who left the index school before graduation in 2003–2006, in almost all cases also due to relocation of the family. Thus, 155 boys and 140 girls remained in this group and graduated just before the intervention was initiated.

As controls we used all other Swedish students (not a sample) who finished 9th grade during the years 2003–2006 (n = 471,926; 241,089 boys and 230,837 girls) and the years 2007–2012 (n = 689,881; 353,439 boys and 336,442 girls). The mean duration of PE per school week in Sweden is 60 min, provided in 1–2 lessons. We must acknowledge, that this duration and frequency in controls are the mean values in Swedish schools, and there could during the nine compulsory school year be differences within the same school between the different grades, between different semesters and also between different schools within the same grades.

### 2.3. End Point Variables—School Grades and Eligibility to Higher Education

Schools in Sweden have to retain grade data for all students for at least 15 years. We collected final elementary grade score card data for each student through the archive of the index school and also registered whether each student was eligible for national upper secondary school or not. The 9th and final elementary school leaving grade report in Sweden includes 16 compulsory school subjects where every subject is graded with the grades Failed (0 points), Passed (10 points), Passed with Distinction (15 points) or Passed with Special Distinction (20 points). The final grade points of these 16 subjects may thus vary from 0 points (p) (no subject with an accepted grade) to 320 p (the highest grade in all subjects). To qualify for national upper secondary school programs the grade Passed is required in each of the subjects Swedish, English and Mathematics. For all other Swedish students the same end point data were retrieved from the Swedish National Agency of Education (Skolverket) [16].

### 2.4. Statistics

We used IBM SPSS Statistics® version 20 for statistical analyses (version 20, IBM, New York, NY, USA). Data are reported as absolute numbers (n), percentages (%), percentage points (pp) or means with 95% confidence intervals (95% CI). No measurement of dispersion is given for national data as these are not based on a sample but include all Swedish children (true values). Group differences were evaluated by Pearson’s chi-square test for eligibility rate, when comparing students in the intervention school graduating 2007–2012 (with the intervention) with students graduating in the same school 2003–2016 (before the intervention) as well as with all Swedish students graduating 2007–2012. We used Student’s unpaired two sample *t*-test when comparing for final grade score means in the two samples of students, those graduating in the intervention school 2007–2012 (with the intervention) with students graduating in the same school 2003–2016 (before the intervention). We finally used Student’s unpaired one sample *t*-test when comparing final grade score means in students in the intervention school graduating 2007–2012 with all Swedish students graduating 2007–2012. We considered *p* < 0.05 to represent a statistically significant difference.

## 3. Results

### 3.1. Comparison between the Index School and All Other Swedish Children Graduating 2003–2006

Before the intervention was initiated a slightly higher proportion of both boys and girls were eligible for upper secondary school in the index school compared to overall Swedish boys and girls (Table 1).

In boys the overall grade points were similar in the intervention and control groups, while girls in the intervention group had significantly higher grades (+21.8 points) then Swedish girls overall (Table 2).

### 3.2. Changes within Groups from 2003–2006 to 2007–2012

Before being compared to after the initiation of the intervention, the proportion of boys eligible for upper secondary school increased in the index school by 7.3 (1.4, 13.2) pp. During the same time frame this proportion decreased in Swedish boys overall by 0.8 pp (Table 1). Among boys, the overall grade points also increased in the index school by 13.3 (3.1, 23.5) points and in Swedish overall boys by 2.7 points (Table 2). In girls, the eligibility rate (Table 1) and overall grades (Table 2) were similar in the two periods both in the index school and overall in Sweden.

### 3.3. Comparison between the Index School and All Other Swedish Children Graduating 2007–2012

With the intervention, both the eligibility rate (+8.3 pp) (Table 1) and the overall grade points (+12.6 points) (Table 2) were higher in boys in the index school compared to all other Swedish children. Among girls, both the eligibility rate (+5.9 pp) (Table 1) and overall grade points (+12.7 points) (Table 2) remained higher in the index school than in all female Swedish students. 

It should also be noted that since the proportion of qualified students (both in boys and girls) decreased in Sweden from 2003–2006 to 2007–2012, the difference between the children in the index school and all other Swedish children increased further with the intervention (Table 1).

## 4. Discussion

In this study we show that boys who had 40 min daily school PA during all nine compulsory school years had higher summarized school grade points and higher qualification rate to upper secondary school than boys who during the nine years had 60 min school PA per week (provided in 1–2 PE lessons). Our results support previous research that suggests that PA is associated with beneficial cognitive achievement [5,11,15,25,26,27,28]. The association between daily PA and an increase by 7.3 percentage points in qualification rate to higher education between 2003–2006 and 2007–2012 is even more impressive, in the perspective that the eligibility rate in Swedish boys has decreased between these two periods.

Some previous research has suggested that there may be need of high intensity PA to improve academic achievement [19]. The cited study promoted 90 min/week of moderate to vigorous physically active academic lessons (3.0 to 6.0 metabolic equivalents (METS), ~10 min each) delivered intermittently throughout the school day. Lessons were usually delivered in the classroom, but were also delivered in school locations such as hallways and outdoors [19]. However, the authors also state in the same article that it is difficult to conclude which exercise dose is required to influence cognitive function and academic achievement [19]. That PA, even at a lower level, also may be beneficial for academic achievement, is supported by data that infer that daily moderate to intense intense PA with activities within the regular school physical education (PE) curriculum, provided as 40 min daily PE classes, was associated with beneficial academic achievement [15]. Since we exposed all children within a school (not only those who chose to participate) to PA activities that were possible for every child to take part in, our study indicates that increased PA is a feasible strategy to improve academic school results in boys on a population-based level.

There are numerous potential hypotheses that may explain the association between increased PA and improved school achievement [15,29,30]. Some studies infer that PA may have direct positive effects on the nervous system by increasing brain volume, blood flow to the brain, synaptic plasticity, as well as promoting formation of nerve cells, all involved in different aspects of perception, cognition, memory, and attention [31,32,33,34]. Other studies suggest that PA has positive effects on psychological parameters such as self-esteem, motivation, social engagement, and communication [12], all of importance for learning outcomes. There are even studies suggesting that inferior motor skills might lead to negative effects in these psychological parameters and delay cognitive development [35,36]. Reports also show an association between higher levels of PA and attention, ability to concentrate in the classroom, and academic achievements [29,37]. We must also emphasize that there could have been other changes, apart from increased PA, during the examination period, changes that we did not register but that still could influence time trends in academic performance. Examples of such changes could be that parents became more aware of the beneficial effects of PA, thereby increasing support to their children to cycle to school and friends, and spend more time with physical activities in their spare time.

Study strengths include the population-based controlled study design, the long-term intervention and the use of endpoint variables relevant to students, parents, teachers and society. Limitations include the low number of individuals in the intervention group, increasing the risk of a type II error, and the absence of comprehensive background data since academic achievement may be affected by other variables such as demographics, motivation, attitudes, extra-curricular activities, access to green spaces, level of spare time PA, socioeconomic status, ethnicity, and parental education. It should be noted that before the intervention was initiated, girls in the intervention school had higher summarized grade points and higher eligibility rate for higher education than Swedish girls in general. The reason for this cannot be explained with our study design, but with this high performance we speculate that the potential to improve was lower in girls than boys. Also, a randomization of the children to the intervention and control groups would have been preferred, but was not accepted by the teachers and parents. Another weakness is that the pre-intervention grades and eligibility to upper secondary school was collected during other years than the data from the intervention cohort. That is, general time trends in grades and eligibility rates not associated with PA may obscure the inferences and our conclusions regarding the effect of daily school physical activity. It would also have been advantageous to have not only data on duration but also the intensity during the PE classes, both on group and individual level, and also how much the children spent on non-organized physical activities.

Finally, we cannot draw any conclusions as regard causality, since there may be a variety of factors associated with the increased physical activity that influenced the school results.

## 5. Conclusions

We conclude that increasing school PA from 60 min per week to 40 min per school day during the nine compulsory school years in boys is associated with improved grade score and higher eligibility rate to secondary school in grade 9. We found no differences in grades or eligibility rates in girls with the intervention. We recommend schools introduce daily PA.

## Figures and Tables

**Table 1:** Numbers and proportion of students who reached eligibility for upper secondary school among boys and girls in the index school and the country of Sweden during years 2003–2006 (before initiation of the intervention in the index school) and 2007–2012 (with intervention in the index school). In Sweden, 241,089 boys and 230,837 girls left grade nine 2003–2006, and 353,439 boys and 336,442 girls 2007–2012.

Reached Eligibility for Upper Secondary School	Students Graduating 2003–2006	Students Graduating 2007–2012	Mean Difference between the Two Periods (pp)
BOYS			
Index school	137 (88.4%)	177 (95.7%)	7.3 (1.4, 13.2)
			*p* < 0.05
Country of Sweden	212,713 (88.2%)	308,850 (87.4%)	−0.8
GIRLS			
Index school	133 (95.0%)	146 (95.4%)	0.4 (−4.5, 5.4)
			*p* = 0.86
Country of Sweden	210,066 (91.0%)	301,216 (89.5%)	−1.5

Data are presented as absolute numbers (n), percentages (%), percentage points (pp) and means with 95% confidence intervals (95% CI). The *p*-value represent the comparison in eligibility rates in the two samples of children from the index school, those that graduated in 2003–2006 with those children that graduated in 2007–2012. Statistically significant group differences are bolded.

**Table 2:** Summarized grade scores for boys and girls in the index school and the country of Sweden during years 2003–2006 (before initiation of the intervention in the index school) and 2007–2012 (with intervention in the index school). In Sweden, 241,089 boys and 230,837 girls left grade nine 2003–2006, and 353,439 boys and 336,442 girls 2007–2012.

Summarized Grade Scores	Students Graduating 2003–2006	Students Graduating 2007–2012	Mean Difference between the Two Periods
BOYS			
Index school	197.7 (189.6, 205.7)	211.0 (204.4, 217.5)	13.3 (3.1, 23.5)
			*p* < 0.05
Sweden	195.7	198.4	2.7
GIRLS			
Index school	239.2 (231.3, 247.1)	233.6 (225.9, 241.3)	−5.6 (−16.6, 5.4)
			*p* = 0.32
Sweden	217.4	220.9	3.5

Data are presented as absolute numbers (n), means with 95% confidence intervals (95% CI) and for country data the actual mean value without 95% CI (since this is not a sample). The *p*-value represents the comparison of summarized grade scores in the two samples of children from the index school, those who graduated in 2003–2006 with those children who graduated in 2007–2012. Statistically significant group differences are bolded.
